# Parental chronic pain in relation to chronic pain in their adult offspring: family-linkage within the HUNT Study, Norway

**DOI:** 10.1186/1471-2458-14-797

**Published:** 2014-08-05

**Authors:** Ragnhild Lier, Tom Ivar Lund Nilsen, Paul Jarle Mork

**Affiliations:** Department of Public Health and General Practice, Norwegian University of Science and Technology (NTNU), N-7491 Trondheim, Norway; Liaison Committee between the Central Norway Regional Health Authority (RHA) and the Norwegian University of Science and Technology (NTNU), N-7491 Trondheim, Norway

**Keywords:** Chronic pain, Epidemiology, Family study, Heritability

## Abstract

**Background:**

Little is known about the association between parental chronic musculoskeletal pain (CMP) and occurrence of CMP in the adult offspring. The main objective of this study was to assess the parent-offspring association of CMP, and also to examine possible modifying effects of age and sex.

**Methods:**

The study includes 11 248 parent-offspring trios from the Norwegian HUNT Study with information on parental CMP obtained in 1995–97 and offspring CMP obtained in 2006–08. Logistic regression was used to calculate adjusted odds ratios (ORs) for offspring CMP associated with parental CMP.

**Results:**

Maternal and paternal CMP was associated with 20-40% increased odds of CMP in sons and daughters. Both sons and daughters had an OR of 1.6 (95% CI 1.4 to 1.9) when both parents reported CMP, compared to when none of the parents had CMP. Restricting the analyses to parental CMP that was associated with limited work ability and leisure time activity did not change the strength of the association. Further, analyses stratified by parental age ±65 years showed no clear difference in the estimated associations, and there was no evidence of interaction for parental sex (P ≥ 0.39) or offspring age ±40 years (P ≥ 0.26).

**Conclusions:**

This large family-linkage study show that maternal and paternal CMP are positively associated with CMP in the adult offspring, irrespective of parental and offspring age, and that the associations are strongest when both parents have CMP. Although the high prevalence of CMP in both parents and offspring suggests that not all cases are clinically relevant, the results suggest that chronic pain has a heritable component.

## Background

Chronic musculoskeletal pain (CMP) is among the leading causes of reduced quality of life and disability in Western countries [1–3]. Several modifiable risk factors have been identified, including physical inactivity [4, 5], obesity [6, 7], and sleep problems [8], although the causal relations are not firmly established. Aggregation of CMP within families also suggests a heritable component [9–11], possibly involving polymorphisms related to catecholamine metabolism [12, 13]. However, while one study using independent pain reports from parents and adolescent offspring found associations in CMP [14], a similar study showed that there was no associations [15]. Hence, there are conflicting results regarding a parent-offspring association of CMP, especially in young offspring. Despite the fact that adult offspring create their own environment outside their family, it has been shown that intergenerational transmissions of lifestyle behaviour manifests in late adolescence and extends into adulthood [16, 17]. Thus, if the development of CMP depends on gene-environment interactions, it is possible that the parent-offspring associations become stronger with increasing offspring age. We are not aware of any population-based study that has examined the parents-offspring association of CMP using offspring data from both early and late adulthood. Moreover, family studies have shown that family aggregation of chronic pain and related conditions is mainly attributable to associations between female relatives [9, 18], while twin studies have shown inconsistent results regarding a sex-specific genetic influence on heritability of chronic pain conditions [19–21]. The independent influence of maternal versus paternal CMP on the occurrence of CMP is still undecided, although the difference between twin studies and other family studies suggest that genetic effects are not sex-dependent whereas environmental influences might be differential between mothers and fathers.

The current study utilizes family linkage data from a large population-based health survey in Norway to investigate both the independent and the combined association of paternal and maternal CMP with occurrence of CMP in the adult offspring. We also examined whether the putative parent-offspring association for CMP interacts with parental and offspring age and sex.

## Methods

### Study population

The HUNT Study is a large population-based health survey conducted in Nord-Trøndelag County, Norway. The study has been carried out in three waves, first in 1984–86 (HUNT1), then in 1995–97 (HUNT2), and last in 2006–08 (HUNT3). At all three waves, all residents aged 20 years and older were invited to participate, and information on lifestyle and health related factors were collected by questionnaires, whereas anthropometric data, blood pressure, and a venous blood sample were obtained at a clinical examination. More detailed information about participation, questionnaires, and procedures in the HUNT study can be read elsewhere [22].

The current study is based on information from HUNT2 and HUNT3 as no information on musculoskeletal pain was obtained at HUNT1. At HUNT2, 93 898 persons were invited to participate and 65 237 (70%) attended the study, whereas 93 860 persons were invited to HUNT3 and 50 807 (54%) chose to participate [23, 24]. The unique personal identification number held by all Norwegian citizens was used to link each participant's record to information from the Family Registry at Statistics Norway, and thus establish a linkage between parents and offspring in the HUNT Study. For the purpose of the present study, we selected all 11 248 parent-offspring trios (i.e., father, mother, and child) with complete information on CMP using parental data from HUNT2 (1995–97) and offspring data from HUNT3 (2006–08).

Participation in the HUNT Study was voluntary and each participant signed a written consent. The study was approved by the Regional Committee for Ethics in Medical Research, (ref.no 2011/1455/REK midt), and carried out according to the Declaration of Helsinki.

### Chronic musculoskeletal pain

The participants were asked to complete a questionnaire that included items on musculoskeletal pain adopted from the Standardized Nordic Questionnaire [25], which has been evaluated and found to have acceptable reliability and validity for upper limb and neck pain, and likely to have a high utility in screening and surveillance [26, 27]. The key question in both HUNT2 and HUNT3 was:” During the last year, have you had pain and/or stiffness in your muscles and joints that lasted for at least three consecutive months?” (response options: “no” and “yes”). We use the term “any CMP” to denote participants who answered “yes” to this question, whereas those who answered “no” formed the reference category for all comparisons. Participants with CMP were also asked to indicate if the pain had led to reduced leisure time activity (response options: “no”, and “yes”) or reduced their work ability (response options: “no”, “to some extent”, “considerably”, or “don’t know”). Participants, who answered “yes” to the question on reduced leisure time activity and “to some extent” or “considerably” on reduced work ability, were classified as having “activity-interfering CMP”.

### Other variables

Standardized measurements of body height (to the nearest centimetre) and body mass (to the nearest kilogram) obtained at the clinical examination were used to calculate body mass index (BMI) as mass divided by the square of height (kg/m^2^).

Leisure time physical activity was assessed from the question: “How much of your leisure time have you been physically active during the last year? (Think of a weekly average for the year. Your commute to work counts as leisure time)”. The participants should report the number of hours of either light (no sweating or heavy breathing) or hard (sweating and heavy breathing) activity using the response options “none”, “less than 1 hour”, “1-2 hours”, and “3 or more hours” for each type of activity. Based on this information, we constructed a new variable with four categories combining information on light and hard activity: 1) “no light or hard activity”, 2) “<3 hours light and no hard activity”, 3) “≥3 hours light and/or <1 hour hard activity”, and 4) “any light and ≥1 hour hard activity”.

Psychological wellbeing was assessed from the question: “Thinking about your life at the moment, would you say that you by and large are satisfied with life, or are you mostly dissatisfied?” The participants were classified into three groups: 1) “satisfied” (response options “very satisfied” and “satisfied”), 2) “somewhat satisfied” (response options “somewhat satisfied”, “neither satisfied nor dissatisfied”, and “quite dissatisfied”), and 3) “dissatisfied” (response options “dissatisfied” and “very dissatisfied”).

### Statistical analysis

Logistic regression was used to estimate odds ratios (ORs) of CMP in offspring associated with maternal and paternal CMP. Since the nature and symptom burden of CMP can differ between younger and older adults [2, 28], and because genetic influence is reported to become less important with increasing age [21], we conducted a stratified analysis by parental age <65 years and ≥65 years. Additionally, a likelihood ratio test was used to examine possible effect modification by offspring age (<40 years and ≥40 years), and also by parental sex. Trios where none of the parents reported CMP defined the reference category in all analyses. All analyses were conducted separately for daughters and sons. Potential confounders were selected after construction of directed acyclic graphs (DAGs) [29] based on a priori knowledge of possible risk factors for CMP. From this procedure, parental characteristics where chosen as possible confounders since they are likely to be associated with both the exposure (i.e., parental CMP) and the outcome (i.e., offspring CMP), whereas offspring characteristics may only be associated with the outcome or act as mediating factors [29]. Moreover, parental and offspring lifestyle factors such as BMI and leisure time physical activity may be highly correlated [30], and factors such as education and psychological well-being are related to pain in both parents and offspring [2]. Thus, the main multivariable models were adjusted for the following paternal and maternal characteristics as potential confounders: age (continuous), BMI ([kg/m^2^], continuous), leisure time physical activity (inactive, low, moderate, high, unknown), education (<10 years, 10-12 years, ≥13 years, unknown), and psychological well-being (satisfied, somewhat satisfied, dissatisfied, unknown). Paternal and maternal CMP were mutually adjusted for when assessing their independent association with offspring CMP by including both as covariates in the regression model. Although not argued for by DAGs, we also assessed potential confounding by the same offspring characteristics in supplementary analyses. Precision of ORs was assessed by 95% confidence interval (CI). All standard errors were adjusted for within-family clustering (i.e., siblings) using the vce (cluster) option in Stata, treating observations between families as independent and within families as dependent, and thus avoiding inflated precision of the estimated associations [31]. To assess possible influence of pain severity, we conducted supplementary analyses of activity-interfering CMP in parents. Since this exposure was partly defined by work ability, these analyses were conducted on trios where both parents were ≤65 years. Finally, to assess if parent-offspring associations are different for more severe CMP we conducted a sensitivity analysis where offspring CMP was restricted to activity-interfering CMP (i.e. pain that caused reduced activity in work and/or leisure time). All statistical tests were two-sided, and all analyses were conducted using Stata for Windows, V.11.0 (StataCorp LP, Texas, USA).

## Results

The study population comprises 11 248 trios including 6 307 daughters and 4 941 sons linked with both their mother and father. Characteristics of the study population are presented in Table 1. The prevalence of any parental CMP at HUNT2 (1995–97) was 56.7% among mothers and 51.4% among fathers while the offspring prevalence of any CMP at HUNT3 (2006–08) was 47.3% among daughters and 39.3% among sons. The prevalence of interfering CMP was somewhat lower (51.4% in mothers, 45.5% in fathers, and 32.2%, and 25.5% in daughters and sons, respectively).

**Table 1:** **Baseline characteristics of 11 248 parent-offspring trios, Nord-Trøndelag Health Study**

	Data from HUNT2 (1995–97)		Data from HUNT3 (2006–08)	
Characteristics	Mother	Father	Daughter	Son
Participants, no.	11 248	11 248	6 307	4 941
Age, mean (SD), years	57.2 (12.2)	60.5 (12.5)	41.1 (11.3)	43.1 (11.1)
Body mass index, mean (SD), kg/m^2^	27.2 (4.5)	26.8 (3.4)	26.1 (4.8)	27.2 (3.8)
Higher education^a^, no. (%)	1 587 (14.1)	1 806 (16.1)	N/A	N/A
Physically inactive^b^, no. (%)	858 (7.6)	856 (7.6)	73 (1.2)	116 (2.4)
Any CMP^c^, no. (%)	6 377 (56.7)	5 783 (51.4)	2 984 (47.3)	1 943 (39.3)
Interfering CMP^d^, no. (%)	5 099 (51.4)	4 567 (45.5)	1 575 (32.2)	1 025 (25.5)

*Abbreviations*: CMP, chronic musculoskeletal pain; HUNT, The Nord-Trøndelag Health Study; SD, standard deviation.

^a^Education ≥13 years.

^b^No sessions with leisure time physical activity.

^c^CMP with duration ≥3 months during the last year at any location.

^d^CMP that interfere with work ability and/or leisure time activity.

Table 2 shows ORs for CMP in daughters and sons associated with any CMP in mothers and fathers, both overall and stratified by parental age ±65 years. The multivariable-adjusted analyses showed that both maternal and paternal CMP were associated with increased odds of offspring CMP, and the ORs were largely similar between the parental age strata. Mean age for offspring, mothers, and fathers in the strata of parental age ≤65 years were 35.4 (standard deviation [SD] 8.5) 49.4 (SD 8.1) and 52.2 (SD 8.2), respectively. In the strata of parental age >65 years the corresponding mean ages were 54.2 (SD 6.4), 72 (SD 4.8), and 75.2 (SD 5.1). In the analyses that included all parents, the ORs for CMP in daughters associated with maternal and paternal CMP were 1.4 (95% CI 1.2 to 1.5) and 1.2 (95% CI 1.1 to 1.3), respectively. The corresponding ORs among sons were 1.3 (95% CI 1.1 to 1.5) associated with maternal CMP and 1.2 (95% CI 1.1 to 1.4) associated with paternal CMP. Although, the difference between mother-offspring and father-offspring association was slightly larger among daughters than among sons, these differences were not statistically significant (P-value, 0.08 in daughters and 0.54 in sons). Correspondingly, we did not observe any statistical interaction (i.e., departure from a multiplicative effect) between parental sex and occurrence of CMP in either daughters (P = 0.97) or sons (P = 0.28). Overall, multivariable adjustment for possible confounders only slightly attenuated the results. The results from supplementary analyses adjusted for offspring characteristics were largely similar to the results presented above. Among daughters the ORs for CMP associated with maternal and paternal CMP were 1.4 (95% CI 1.2 to 1.5) and 1.2 (95% CI 1.1 to 1.3), and the corresponding ORs among sons were 1.2 (95% CI 1.1 to 1.4), and 1.2 (95% CI 1.1 to 1.3), respectively.

**Table 2:** **Odds ratios for offspring chronic musculoskeletal pain (CMP) associated with any maternal or paternal CMP**

	Any maternal CMP		Any paternal CMP		P-value
	No	Yes	No	Yes	Difference^c^
Daughters					
All parents					
Cases/non-cases	1 110/1 599	1 834/1 684	1 315/1 685	1 629/1 598	
Age-adjusted OR^a^ (95% CI)	1.0	1.6 (1.4 to 1.7)	1.0	1.3 (1.1 to 1.4)	0.003
Multivariably-adjusted OR^b^ (95% CI)	1.0	1.4 (1.2 to 1.5)	1.0	1.2 (1.1 to 1.3)	0.080
Both parents ≤65 years					
Cases/non-cases	631/1 193	995/1 151	699/1 206	927/1 138	
Age-adjusted OR^a^ (95% CI)	1.0	1.6 (1.4 to 1.8)	1.0	1.3 (1.2 to 1.5)	0.044
Multivariably-adjusted OR^b^ (95% CI)	1.0	1.4 (1.2 to 1.6)	1.0	1.2 (1.1 to 1.4)	0.138
Both parents >65 years					
Cases/non-cases	349/272	547/320	398/315	598/277	
Age-adjusted OR^a^ (95% CI)	1.0	1.3 (1.1 to 1.6)	1.0	1.4 (1.2 to 1.7)	0.629
Multivariably-adjusted OR^b^ (95% CI)	1.0	1.3 (1.0 to 1.6)	1.0	1.4 (1.1 to 1.7)	0.612
Sons					
All parents					
Cases/non-cases	736/1 370	1 166/1 582	868/1 529	1 034/1 423	
Age-adjusted OR^a^ (95% CI)	1.0	1.3 (1.2 to 1.5)	1.0	1.2 (1.1 to 1.4)	0.355
Multivariably-adjusted OR^b^ (95% CI)	1.0	1.3 (1.1 to 1.5)	1.0	1.2 (1.1 to 1.4)	0.542
Both parents ≤65 years					
Cases/non-cases	367/852	576/956	402/938	541/870	
Age-adjusted OR^a^ (95% CI)	1.0	1.3 (1.1 to 1.6)	1.0	1.4 (1.2 to 1.6)	0.792
Multivariably-adjusted OR^b^ (95% CI)	1.0	1.3 (1.1 to 1.5)	1.0	1.3 (1.1 to 1.6)	0.756
Both parents >65 years					
Cases/non-cases	261/364	401/402	321/415	341/351	
Age-adjusted OR^a^ (95% CI)	1.0	1.3 (1.1 to 1.6)	1.0	1.2 (0.9 to 1.5)	0.510
Multivariably-adjusted OR^b^ (95% CI)	1.0	1.4 (1.1 to 1.7)	1.0	1.3 (1.0 to 1.6)	0.587

*Abbreviations*: CI, confidence interval; OR, odds ratio.

^a^Adjusted for parental age (continuous) in HUNT2, and mutually adjusted for maternal and paternal CMP.

^b^Adjusted for factors in ^a^ and parental factors in HUNT2; body mass index ([kg/m^2^] continuous), leisure time physical activity (inactive, low, moderate, high, unknown), psychological well-being (satisfied, somewhat satisfied, dissatisfied, unknown), and education (<10 years, 10–12 years, ≥13 years, unknown).

^c^P-value for the estimated difference between mother-offspring and father-offspring associations.

Table 3 shows ORs for offspring CMP associated with a combined variable of paternal and maternal CMP. Compared to the reference group of no CMP in any of the parents, the OR for CMP in offspring was 1.6 (95% CI 1.4 to 1.9) in both sons and daughters if both parents reported any CMP. Moreover, if only mothers reported any CMP, the OR for offspring CMP was 1.4 (95% CI 1.2 to 1.6) in daughters and 1.4 (95% CI 1.2 to 1.7) in sons. Correspondingly, CMP in only fathers was associated with an OR of 1.2 (95% CI 1.0 to 1.4) in daughters and 1.3 (95% CI 1.1 to 1.6) in sons. Stratified analyses according to offspring age (±40 years) showed no large difference in the parent-offspring associations (data not shown). CMP in both parents was associated with an OR of 1.7 (95% CI 1.4 to 2.2) among daughters <40 years and 1.5 (95% CI 1.2 to 1.8) among daughters ≥40 years. Among sons, the corresponding ORs were 1.7 (95% CI 1.3 to 2.3) and 1.5 (95% CI 1.2 to 1.9), respectively. A likelihood-ratio test of the interaction between parental CMP and offspring age gave P-values of 0.18 in daughters and 0.94 in sons.

**Table 3:** **Odds ratio for offspring chronic musculoskeletal pain (CMP) associated with any parental CMP**

	No CMP	Any Maternal CMP	Any Paternal CMP	Any CMP in both parents
Daughters				
Cases/non-cases	535/884	780/801	575/715	1 054/883
Age-adjusted OR^a^ (95% CI)	1.0	1.6 (1.4 to 1.8)	1.3 (1.1 to 1.5)	1.9 (1.7 to 2.3)
Multivariably-adjusted OR^b^ (95% CI)	1.0	1.4 (1.2 to 1.6)	1.2 (1.0 to 1.4)	1.6 (1.4 to 1.9)
Sons				
Cases/non-cases	367/785	501/744	369/585	665/838
Age-adjusted OR^a^ (95% CI)	1.0	1.5 (1.2 to 1.7)	1.4 (1.1 to 1.6)	1.7 (1.4 to 2.0)
Multivariably-adjusted OR^b^ (95% CI)	1.0	1.4 (1.2 to 1.7)	1.3 (1.1 to 1.6)	1.6 (1.4 to 1.9)

*Abbreviations*: *CI*, confidence interval; *OR*, odds ratio.

^a^Adjusted for parental age (continuous) in HUNT2.

^b^Adjusted for parental factors in HUNT2; age (continuous), body mass index ([kg/m^2^] continuous), leisure time physical activity (inactive, low, moderate, high, unknown), psychological well-being (satisfied, somewhat satisfied, dissatisfied, unknown), and education (<10 years, 10–12 years, ≥13 years, unknown).

In a supplementary analysis (Table 4) we examined if pain severity could influence these associations by restricting the exposure to activity-interfering CMP in parents aged ≤65 years. The presence of interfering CMP in either mother or father was associated with 30-50% increased odds of CMP in the offspring. When both parents reported interfering CMP, the OR was 1.9 (95% CI 1.5 to 2.4) among daughters and 1.6 (95% CI 1.3 to 2.2) among sons. These associations were slightly strengthened in a sensitivity analysis restricting the outcome to activity-interfering CMP in offspring, with an OR of 2.4 (95% CI 1.8 to3.1) among daughters, and 1.8 (95% CI 1.2 to 2.5) among sons (data not shown).

**Table 4:** **Odds ratio for offspring chronic musculoskeletal pain (CMP) associated with activity-interfering CMP in parents aged ≤65 years**

		Activity-interfering CMP		
	No CMP	Maternal	Paternal	Both parents
Daughters				
Cases/non-cases	297/675	305/407	261/404	407/373
Age-adjusted OR^a^ (95% CI)	1.0	1.7 (1.4 to 2.1)	1.5 (1.2 to 1.8)	2.5 (2.0 to 3.0)
Multivariably-adjusted OR^b^ (95% CI)	1.0	1.4 (1.1 to 1.7)	1.3 (1.0 to 1.6)	1.9 (1.5 to 2.4)
Sons				
Cases/non-Cases	169/486	190/355	160/283	219/324
Age-adjusted OR^a^ (95% CI)	1.0	1.5 (1.2 to 2.0)	1.6 (1.3 to 2.1)	1.9 (1.5 to 2.5)
Multivariably-adjusted OR^b^ (95% CI)	1.0	1.3 (1.3 to 1.8)	1.5 (1.1 to 1.9)	1.6 (1.3 to 2.2)

*Abbreviations*: *CI*, Confidence interval; *OR*, Odds ratio.

^a^Adjusted for parental age (continuous) in HUNT2.

^b^Adjusted for parental factors in HUNT2; age (continuous), body mass index ([kg/m^2^] continuous), leisure time physical activity (inactive, low, moderate, high, unknown), psychological well-being (satisfied, somewhat satisfied, dissatisfied, unknown), and education (<10 years, 10–12 years, ≥13 years, unknown).

## Discussion and conclusion

In this large population-based family linkage study we found that both paternal and maternal CMP was associated with increased occurrence of CMP in the adult offspring, and this association was particularly strong when CMP was present in both parents. Restricting the analyses to CMP that interfered with work ability and leisure time activity did not materially change the odds of CMP in the offspring. Further, we found no evidence that offspring age or parental sex modified the parent-offspring association of CMP.

It is well established that independent pain reports from parents and offspring are necessary to achieve acceptable validity in family-linkage studies [32]. We are only aware of two previous studies that have investigated parent-offspring associations within the same study population using independent pain reports from parents and offspring [14, 15]. Both studies investigated the influence of parental pain on occurrence of chronic pain in young, adolescent offspring. While Hoftun and colleagues [14] reported a moderate parent-offspring association for chronic pain, Jones and colleagues [15] found no association between parent and offspring pain. Based on the findings in the latter study the authors suggested that pain behaviour is not learned, but is rather attributable to individual factors and the social environment. However, it is also possible that the parent-offspring association of pain changes with increasing age in the offspring. It has been shown that the transmission of lifestyle behaviour across generations manifests itself more strongly in late adolescence and extends into adulthood [16, 17] and may encompass risk factors for CMP such as physical inactivity and obesity [7, 16, 17]. Thus, we hypothesised that offspring and parents would become more alike with respect to CMP after the offspring approach middle-age compared to younger adulthood. However, we found no evidence of an interaction between offspring age (<40 years versus ≥40 years) and occurrence of offspring CMP, and stratified analyses gave largely similar associations.

Previous family linkage studies and twin studies have provided conflicting results regarding the effect of sex on heritability of CMP. While some studies have reported sex-dependent associations [9, 10, 18], large-scale twin studies have shown minor [20, 21] or no [19] sex-specific genetic influence on chronic pain conditions. In the present study, there was no clear difference between the maternal-offspring and paternal-offspring associations of CMP, and we found no evidence of interaction with parental sex and occurrence of CMP in sons and daughters.

The current results suggest a stronger parent-offspring association if both parents report CMP than if only one parent have CMP. Thus, one may speculate that the occurrence of CMP in the adult offspring is strongly influenced by genetic factors. Conversely, it has been suggested that children of parents who display pain behavior adopt similar behaviors and are also more likely to report pain than their peers [32, 33]. However, our data did not allow us to decide the relative contribution of genetic and environmental factors to CMP. It has been suggested that inheritance of CMP is more pronounced in severe and disabling pain conditions with widespread pain, such as fibromyalgia [9, 34], compared to conditions with milder and more localized symptoms [10]. We had no information about pain intensity in the current study, but supplementary analysis restricted to both parents and offspring with CMP that interfered with work ability and leisure time activity gave largely similar results as the main analyses. Although our results are not directly comparable with previous studies regarding the impact of symptom severity on parent-offspring associations, they indicate no different associations for CMP that limits activity and non-interfering CMP. Musculoskeletal disorders are the most frequent cause of sick leave and disability in Norway [35]. However, many people do not consult their doctor with their complaints [36], and it is likely that the definition of CMP used in the current study embrace a large variation of severity levels that could be relevant in a public health perspective irrespective of their health seeking behaviour.

There are several strengths to the current study, including the large number of parent-offspring trios, the population-based nature of the data, and the ability to link family members using the Family Registry at Statistics Norway. In contrast to previous studies using extended families [9, 10] or family history of pain reported by the young offspring [18], we investigated the parent-offspring association using independent pain reports from parents at HUNT2 (1995–97) and from adult offspring at HUNT3 (2006–08). Another strength of this study was the ability to adjust for parental characteristics associated with CMP, including age [2], BMI [6, 7, 37, 38], leisure time physical activity [4, 7], psychological well-being [8, 39], and education [10, 40]. It may be argued that offspring characteristics are more likely to be associated with offspring CMP, but results from additional analyses adjusted for offspring characteristics were similar to those adjusted for parental characteristics. However, as in all observational studies, residual confounding due to unmeasured and unknown factors cannot be ruled out. Although we are not able to decide the relative contribution of genetic and environmental factors or possible epigenetic effects [41, 42], the sparse attenuations in the results after adjusting for potential confounders might indicate that parental lifestyle, psychological factors, and socioeconomic status have minor influence on the parent-offspring association of CMP. This is in agreement with a recent study on adolescents from the same population [14]. Although self-reported information on CMP, leisure time physical activity, education, and psychological well-being could be prone to misclassification [43], it is not likely that such misclassification is differential between pain-afflicted and pain-free individuals. Nevertheless, when generalizing these results to a broader population it should be noted that the trios included in the current study may constitute a selected sample in terms of family structure and health status. The participation rate was substantially lower at HUNT3 (54%) than at HUNT2 (71%), and non-participants in HUNT3 are reported to have less musculoskeletal symptoms, lower BMI, and lower socioeconomic status than participants [24].

In conclusion, this family-linkage study shows that CMP in mothers and fathers was consistently associated with higher occurrence of CMP in the adult offspring, especially if both parents reported CMP. These associations persisted also after adjusting for parental or offspring characteristics and they were not modified by offspring age. Moreover, restricting the analyses to parental activity-interfering CMP did not change the strength of the associations. The high prevalence of CMP in both parents and offspring, also for activity interfering CMP, suggests that not all cases are clinically relevant. Nevertheless, despite that the relative contribution of genetic and environmental factors could not be decided in this study, our data clearly demonstrate family clustering that is in agreement with a heritable component of CMP.

## Abbreviations

BMI: Body mass index
CI: Confidence interval
CMP: Chronic musculoskeletal pain
OR: Odds ratio.
